# Ubiquitination at the lysine 27 residue of the Parkin ubiquitin-like domain is suggestive of a new mechanism of Parkin activation

**DOI:** 10.1093/hmg/ddac064

**Published:** 2022-03-21

**Authors:** Jun-Yi Liu, Tsuyoshi Inoshita, Kahori Shiba-Fukushima, Shigeharu Yoshida, Kosuke Ogata, Yasushi Ishihama, Yuzuru Imai, Nobutaka Hattori

**Affiliations:** Department of Neurology, Juntendo University Graduate School of Medicine, Tokyo 113-8421, Japan; Department of Neurodegenerative and Demented Disorders, Juntendo University Graduate School of Medicine, Tokyo 113-8421, Japan; Department of Drug Development for Parkinson’s Disease, Juntendo University Graduate School of Medicine, Tokyo 113-8421, Japan; Department of Molecular and Cellular BioAnalysis, Graduate School of Pharmaceutical Sciences, Kyoto University, Kyoto 606-8501, Japan; Department of Molecular and Cellular BioAnalysis, Graduate School of Pharmaceutical Sciences, Kyoto University, Kyoto 606-8501, Japan; Department of Molecular and Cellular BioAnalysis, Graduate School of Pharmaceutical Sciences, Kyoto University, Kyoto 606-8501, Japan; Department of Neurology, Juntendo University Graduate School of Medicine, Tokyo 113-8421, Japan; Department of Research for Parkinson’s Disease, Juntendo University Graduate School of Medicine, Tokyo 113-8421, Japan; Department of Neurology, Juntendo University Graduate School of Medicine, Tokyo 113-8421, Japan; Department of Neurodegenerative and Demented Disorders, Juntendo University Graduate School of Medicine, Tokyo 113-8421, Japan; Department of Drug Development for Parkinson’s Disease, Juntendo University Graduate School of Medicine, Tokyo 113-8421, Japan; Department of Research for Parkinson’s Disease, Juntendo University Graduate School of Medicine, Tokyo 113-8421, Japan

## Abstract

The mitochondrial kinase PTEN-induced kinase 1 (PINK1) and cytosolic ubiquitin ligase (E3) Parkin/PRKN are involved in mitochondrial quality control responses. PINK1 phosphorylates ubiquitin and the Parkin ubiquitin-like (Ubl) domain at serine 65 and promotes Parkin activation and translocation to damaged mitochondria. Upon Parkin activation, the Ubl domain is ubiquitinated at lysine (K) 27 and K48 residues. However, the contribution of K27/K48 ubiquitination toward Parkin activity remains unclear. In this study, ubiquitination of K56 (corresponding to K27 in the human), K77 (K48 in the human) or both was blocked by generating *Drosophila* Parkin (dParkin) mutants to examine the effects of Parkin Ubl domain ubiquitination on Parkin activation in *Drosophila*. The dParkin, in which K56 was replaced with arginine (dParkin K56R), rescued pupal lethality in flies by co-expression with PINK1, whereas dParkin K77R could not. The dParkin K56R exhibited reduced abilities of mitochondrial fragmentation and motility arrest, which are mediated by degrading Parkin E3 substrates Mitofusin and Miro, respectively. Pathogenic dParkin K56N, unlike dParkin K56R, destabilized the protein, suggesting that not only was dParkin K56N non-ubiquitin-modified at K56, but also the structure of the Ubl domain for activation was largely affected. Ubiquitin attached to K27 of the Ubl domain during PINK1-mediated Parkin activation was likely to be phosphorylated because human Parkin K27R weakened Parkin self-binding and activation *in trans*. Therefore, our findings suggest a new mechanism of Parkin activation, where an activation complex is formed through phospho-ubiquitin attachment on the K27 residue of the Parkin Ubl domain.

## Introduction

Mutations in the genes encoding Parkin/PRKN and PTEN-induced kinase 1 (PINK1) cause selective degeneration of the midbrain dopaminergic neurons in autosomal recessive juvenile parkinsonism (1,2). Parkin, a 465-residue ubiquitin ligase (E3), comprises a ubiquitin-like (Ubl) domain, a RING0 domain, a RING1 domain that binds to ubiquitin-conjugating enzymes (E2s), an In-Between-RING (IBR) domain and a RING2 domain that mediates the enzyme catalytic activity (3–5). The *PINK1* gene codes for a mitochondrial serine/threonine protein kinase. A series of studies have demonstrated that PINK1 and Parkin cooperatively regulate mitochondrial quality control by removing damaged mitochondria (6–8). When the mitochondrial membrane potential is reduced, PINK1 is accumulated and activated at the mitochondrial outer membrane, which recruits Parkin from the cytoplasm to the mitochondria and activates Parkin E3. Parkin translocated to the mitochondria ubiquitinates and degrades mitochondrial proteins, such as Mitofusin and Miro (9,10).

Previously, we showed that during Parkin translocation, PINK1 phosphorylates Parkin at serine 65 (S65) in the Ubl domain to promote mitophagy in cultured cells (11). We also demonstrated dParkin Ubl domain phosphorylation in *Drosophila*, which occurs at S94, boosting E3 activity. In contrast, the replacement of S94 with alanine (A) inhibits dParkin E3 activity, which has been confirmed by the estimation of mitochondrial morphology and distribution in dopaminergic neurons (12).

Parkin binds to phospho-S65 monoubiquitin and polyubiquitin (13–17), which primes PINK1-mediated phosphorylation of the Parkin Ubl domain (14,18,19). A study using hydrogen-deuterium exchange mass spectrometry revealed that an amino acid region spanning from the 20th to 30th residue in the human Parkin Ubl domain containing the lysine 27 (K27) residue most actively shows hydrogen-deuterium exchange after the binding of phospho-ubiquitin and subsequent Ubl domain phosphorylation by PINK1 (5). In contrast, E2 binding to Parkin protects this amino acid region in the Ubl domain from deuterium replacement, although this Ubl domain region is expected to be exposed to the solvent (5). This suggests that the 20–30 amino acid region in the Ubl domain is subject to modification after Parkin activation. Another study revealed that upon Parkin activation, the K27 and K48 residues of the Ubl domain are ubiquitinated (20). However, this study failed to elucidate the significant effects of K27 or K48 replacement with arginine (R) and S65 replacement with A upon Parkin activation and mitochondrial translocation (20). Although the potential ubiquitin modification of the Ubl domain during Parkin activation is a reproducible phenomenon, its physiological significance is not well understood.

A previous study described that the pathogenic K27 → asparagine (N) modification in Parkin found in autosomal recessive juvenile parkinsonism has no effects on Parkin auto-ubiquitination (21,22). In contrast, two other studies reported that the K27N mutant, but not K48A, affects ubiquitin chain assembly during Parkin activation and mitophagy (17,23). Thus, the extent to which ubiquitination at K27 or K48 affects Parkin activation remains poorly defined and needs to be clarified.

In this study, using *Drosophila* phenotype assays, we analyzed the effects of mutations at the K27 and K48 residues in the Ubl domain on Parkin E3 activity. Our findings provide insights into a potentially new mechanism of Parkin activation, which may contribute to our understanding of the sophisticated PINK1/Parkin-mediated mitochondrial quality control mechanism and facilitate the development of therapeutic strategies for Parkin-targeted mitochondria-related diseases.

## Results

### The K27 residue in the Parkin Ubl domain is involved in Parkin activation

**Figure 1:**
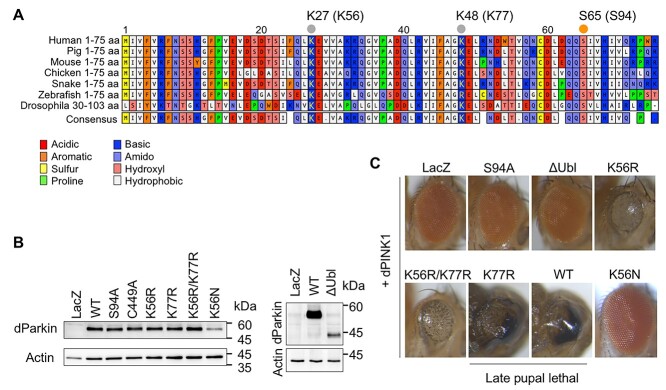
Lysine 56 (K56) mutation weakens dParkin activation in *Drosophila* eyes. (**A**) Sequence alignment of the Ubl domain of Parkin from a variety of species generated by ClustalW. K27 (K56 in *Drosophila*), K48 (K77 in *Drosophila*) and S65 (S94 in *Drosophila*) residues are shown by dots. (**B**) Expression of dParkin (WT, S94A, C449A, K56R, K77R, K56R/K77R, K56N and ∆Ubl) or β-galactosidase (LacZ) in the developing eye cells of *Drosophila* was evaluated using the *GMR-GAL4* driver. LacZ and WT dParkin were used as negative and positive controls, respectively. (**C**) Late pupal lethal phenotype resulting from the genetic interaction between *Drosophila PINK1* (*dPINK1*) and WT *dParkin* and K77R *dParkin*, but not K56R/N *dParkin*. *PINK1* and the indicated *dParkin* genes were co-expressed in the *Drosophila* eyes at 18°C using the *GMR-GAL4* driver. WT and K77R were imaged with their heads removed from their shells.

Our previous phospho-proteomic analysis of Parkin-mediated mitophagy revealed ubiquitination of Parkin at K27/K48 in the Parkin Ubl domain in addition to phosphorylation of Parkin and ubiquitin (Supplementary Material, Fig. S1) (11,15), which has also been reported in other studies (17,20). The K27 (K56 in *Drosophila*) residue in the Ubl domain is perfectly conserved among species, whereas the K48 (K77 in *Drosophila*) residue is moderately conserved (Fig. 1A). To determine the effects of K27/K48 ubiquitination on Parkin activation, we generated *Drosophila* expressing K56R/K77R double mutant Parkin and K56R or K77R single mutant Parkin (Fig. 1B and Supplementary Material, Fig. S2A).

As described in our previous study reporting *Drosophila* expressing phosphomimetic Parkin (dParkin S94E) (12), Parkin overactivation is neurotoxic such that overexpression of wild-type (WT) *Drosophila* Parkin (dParkin) along with *Drosophila PINK1* in the insect eyes resulted in lethal pupa (Fig. 1C). In contrast, overexpression of WT dParkin alone or dParkin S94A (corresponding to human Parkin S65A) with PINK1 produced a normal eye phenotype (Fig. 1C). Deletion of the Ubl domain from dParkin also showed null effects in the eyes, even in the presence of *PINK1* expression (Fig. 1C). In this line, dParkin with K56/K77R or K56R, but not dParkin K77R, partially suppressed the lethal phenotype, leading to the emergence of adult flies with degenerated eyes. These genetic analyses suggest that the ubiquitination of K56, but not K77 residue, contributes to Parkin E3 activation. Surprisingly, dParkin K56N, which corresponds to human Parkin with the K27N pathogenic mutation, exhibited no eye degeneration (Fig. 1C). This null phenotype of dParkin K56N appeared to be attributed to both protein instability and non-ubiquitination (Fig. 1B).

### Blocking of ubiquitination at K27 in the Ubl domain reduces Parkin mitochondrial regulation *in vivo*

**Figure 2:**
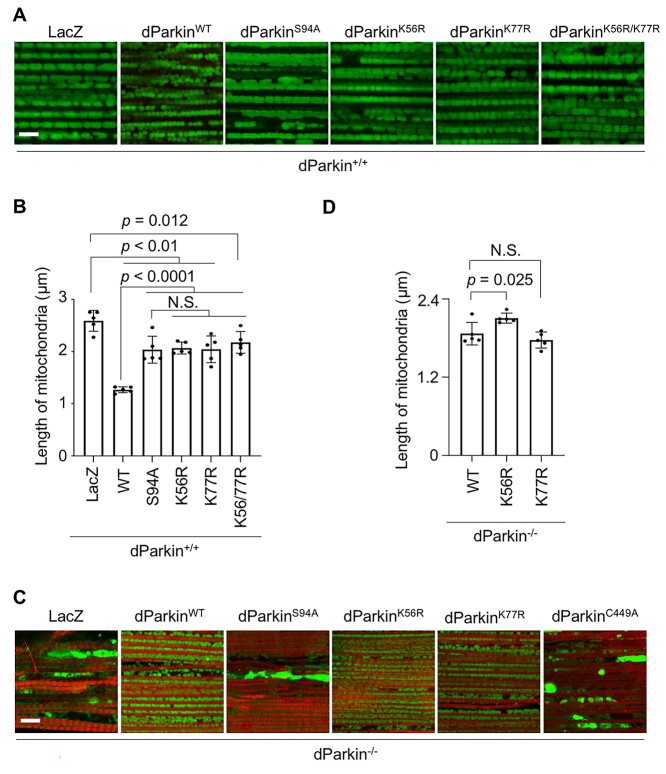
dParkin K56R weakens mitochondrial regulatory activity in *Drosophila* muscles. (**A**) Representative images of the longitudinally sectioned thorax tissues of 3-day-old flies expressing the indicated genes under the *dParkin*^+/+^ genetic background using the *MHC-GAL4* driver. Muscular mitochondria (green) were visualized by mitoGFP. Scale bar = 4 μm. (**B**) Graph showing the length of the long axis of the muscle mitochondria obtained from five flies as in (A). Each data point (average length from 50 mitochondria in each fly) and standard deviation are shown as a dot and error bar, respectively. Comparisons were performed using Tukey’s test. (**C**) Representative images of thoracic mitochondria (green) of *dParkin*^−/−^ flies expressing the indicated genes using the *MHC-GAL4* driver. Myofibrils were counterstained with TRITC-phalloidin (red). Scale bar = 8 μm. (**D**) Graph showing the length of the long axis of the muscle mitochondria obtained from five flies (*n* = 50 mitochondria in each fly). Comparisons were performed using Dunnett’s test (versus WT). N.S., not significant.

Mitochondrial morphology reflects dParkin E3 activity because the mitochondrial fusion GTPase Mitofusin is a ubiquitination substrate of dParkin (12). The overexpression of WT dParkin in the WT genetic background (i.e. in the presence of endogenous dParkin) led to mitochondrial fragmentation in indirect flight muscles, whereas dParkin S94A did not affect mitochondrial length compared to WT dParkin (Fig. 2A and B). Similar to S94A, dParkin K56R/K77R, K56R or K77R had weaker effects on mitochondrial fragmentation than WT dParkin (Fig. 2A and B). The levels of Mitofusin as well as those of mitochondrial respiratory proteins, including mitochondrial complex I subunit NDUFS3 and complex V subunit ATP5A, were not significantly different between flies expressing WT dParkin and the Ubl domain mutants, namely S94A, K56R/K77R, K56R and K77R, in the thorax muscle tissue under the *dParkin* (*park*)-deficient genetic background (Supplementary Material, Fig. S2B and C). These results suggested that the levels of mitochondrial proteins, which would be altered by the Ubl domain mutations, were below the detection limit.

WT dParkin overexpression completely rescued the mitochondrial degeneration caused by dParkin loss, whereas mutant dParkin S94A and E3-dead dParkin C449A did not (Fig. 2C). In the absence of endogenous dParkin, dParkin K56R, but not dParkin K77R, produced mitochondria larger in length than that produced by WT dParkin (Fig. 2C and D), suggesting that the efficiency of Mitofusin degradation was reduced by K56R mutation.

**Figure 3:**
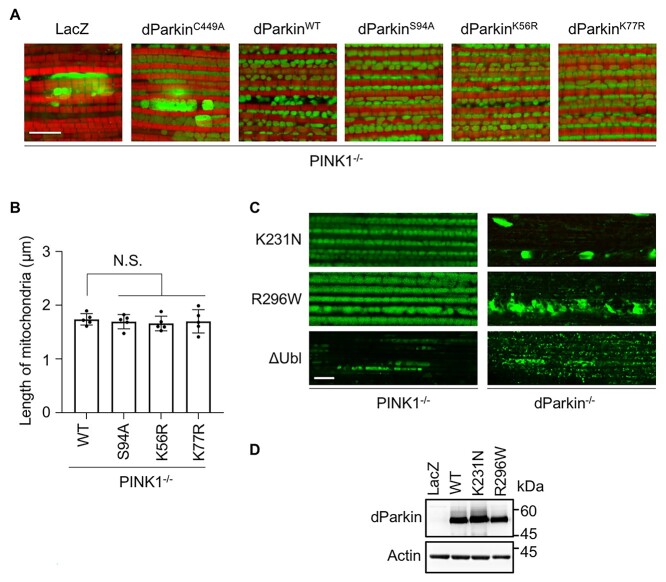
dParkin Ubl rescues the mitochondrial degeneration in *PINK1*-deficient flies. (**A**) Thorax mitochondrial images of *PINK1^−/−^* flies expressing the indicated genes. Scale bar = 8 μm. (**B**) The length of the long axis of the muscle mitochondria from five flies (*n* = 50 mitochondria in each fly) is represented using a graph. The comparison was performed using the Dunnett test (versus WT); N.S., not significant. (**C**) Representative images of thoracic mitochondria (green) of *PINK1^−/−^* or *dParkin*^−/−^ flies expressing the indicated genes using the *MHC-GAL4* driver. Scale bar = 4 μm. (**D**) Expression of dParkin missense mutants used in (C). Expression of dParkin in the thorax muscle of normal *w^−^* flies evaluated using the *MHC-GAL4* driver. LacZ and WT dParkin were used as negative and positive controls, respectively.

In the *PINK1*-deficient background, the E3 activity of dParkin S94A (assessed by the mitochondrial length) was similar to that of WT, as we previously reported (12), and the other Ubl mutants were not different from WT dParkin (Fig. 3A and B). Overexpression of E3-dead dParkin C449A failed to rescue the *PINK1^−/−^* mitochondrial phenotype (Fig. 3A). In contrast, the disease-associated dParkin mutants, K231N (K211N in human) and R296W (R275W in human), rescued mitochondrial degeneration in *PINK1*-deficient flies, but not *dParkin*-deficient flies (Fig. 3C and D). However, dParkin lacking the Ubl domain (∆Ubl) failed to rescue the mitochondrial degeneration in *PINK1*-deficient and *dParkin*-deficient flies (Fig. 3C). These results suggest that both the dParkin Ubl domain and E3 activity, even if it is weak, are required for endogenous dParkin activation *in trans*.

**Figure 4:**
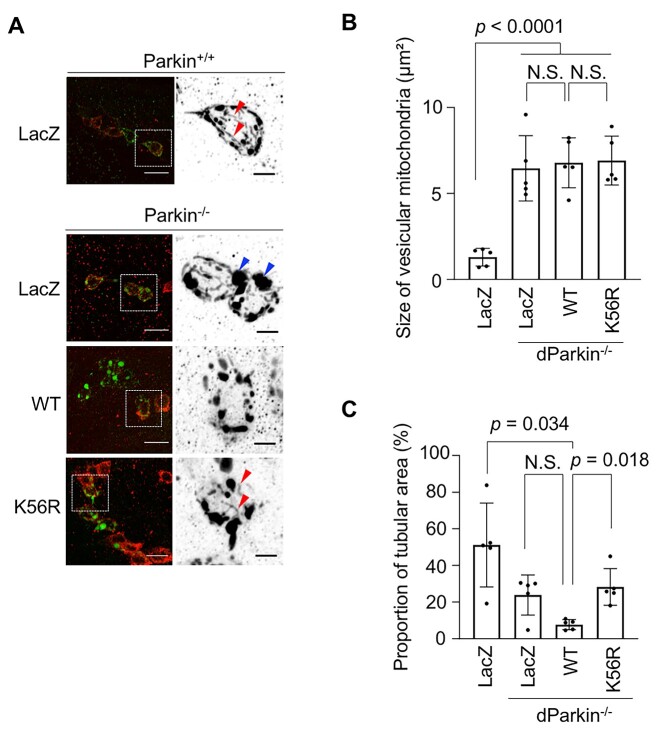
dParkin K56R increases the proportion of tubular mitochondria in dopaminergic neurons**.** (**A**) LacZ, WT dParkin or dParkin K56R was expressed together with mitoGFP in dopaminergic neurons in *dParkin^−/−^* flies using the *TH-GAL4* driver. In the left column, mitochondria and cell bodies of the *proto*cerebral posterior lateral *1* (PPL1) cluster dopaminergic neurons were visualized using mitoGFP (green) and anti-tyrosine hydroxylase staining (red), respectively. In the right column, the signal intensity of the mitochondrial single channel in enlarged areas of the dashed boxes has been inverted for clarity. Red and blue arrowheads indicate tubular and large vesicular (>2 μm) mitochondria, respectively. Scale bars = 10 μm (left) and 3 μm (right). (**B**) Bar graph with data points represents the area of vesicular mitochondria in the PPL1 tyrosine hydroxylase positive-dopaminergic neurons. Each data point indicates the average area of vesicular mitochondria in a single neuron (*n* = 5–9 neurons from five flies) in each fly. The comparison was performed using the Dunnett test. (**C**) The graph represents the proportion of tubular mitochondria in the PPL1 tyrosine hydroxylase-positive neurons. Each data point indicates the average proportion of tubular mitochondria in a single neuron (*n* = 5–9 neurons from five flies) in each fly. Welch ANOVA with Steel tests was performed for multiple comparisons.

Given that dParkin K56R showed apparently impaired E3 activity with respect to both the eye phenotype and mitochondrial morphology, we next evaluated the effects of dParkin K56R on neuronal mitochondria. The morphology of mitochondria in dopaminergic neurons of the adult *Drosophila* brain was analyzed through deconvolution. In *dParkin^−/−^* cells, large, aggregated mitochondria have been reported (24), whereas WT dParkin expression in *dParkin^−/−^* cells in this study resulted in mild mitochondrial fragmentation, thereby reducing the amount of aggregated mitochondria (Fig. 4A and B). This phenomenon occurs likely because of the degradation of Mitofusin by Parkin (12). In this setting, expression of dParkin K56R in *dParkin^−/−^* cells caused an increased population of tubular mitochondria compared with that in cells expressing WT dParkin (Fig. 4C), which again suggests that Parkin E3 activity is reduced because of K56R mutation.

**Figure 5:**
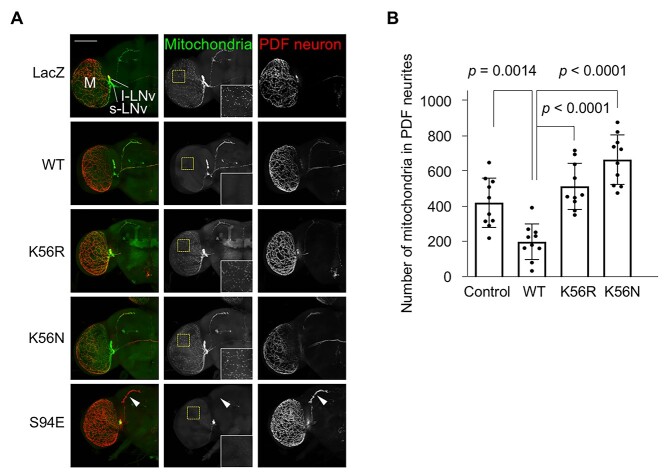
dParkin K56R alters mitochondrial distribution in PDF-expressing neurons. (**A**) LacZ (control), WT dParkin, dParkin K56R, dParkin K56N and dParkin S94E were expressed together with mitoGFP in PDF-positive small lateral ventral clock (s-LNv) and large lateral ventral clock (l-LNv) neurons using the *PDF-GAL4* driver. Lines indicate the cell bodies of s-LNv and l-LNv neurons. M, medulla of the optic lobe. Mitochondria and the neuronal processes of PDF-expressing neurons were observed by labeling with mitoGFP (green) and staining with anti-PDF (red), respectively. Enlarged areas of the yellow-dashed boxes are shown in the insets. Arrowheads indicate s-LNv terminals lacking mitochondria. Scale bar = 100 μm. (**B**) Bar graph with data points indicates the number of mitochondria colocalized with the PDF-positive neurites extending in the medulla (*n* = 10 flies). Multiple comparison was performed by Dunnett’s test (versus WT dParkin).

Parkin negatively regulates mitochondrial motility by modulating the levels of Miro, a mitochondrial Rho GTPase (10,25). The distribution of neuronal mitochondria was evaluated using pigment-dispersing factor (PDF)-expressing neurons (Fig. 5), which contain two neuron clusters (small and large lateral ventral clock neurons), because their neuronal processes are easily observed in whole-mount brain sections (26). Ectopic expression of WT dParkin in PDF-expressing neurons resulted in the disappearance of mitochondria in the neurites of the medulla, compared with that of control LacZ and the pathogenic dParkin K56N (Fig. 5A and B). dParkin S94E expression further enhanced this phenotype observed with WT dParkin such that mitochondria of small lateral ventral clock neuronal terminals also disappeared, leaving in the cell bodies (Fig. 5A). In contrast, dParkin K56R led to a partial mitochondrial recovery in the neurites of the medulla (Fig. 5A and B). These results supported that K56 (K27 in human) is one of the key residues involved in Parkin activation. 

### Phospho-ubiquitination at K27 facilitates Parkin activation *in trans*

**Figure 6:**
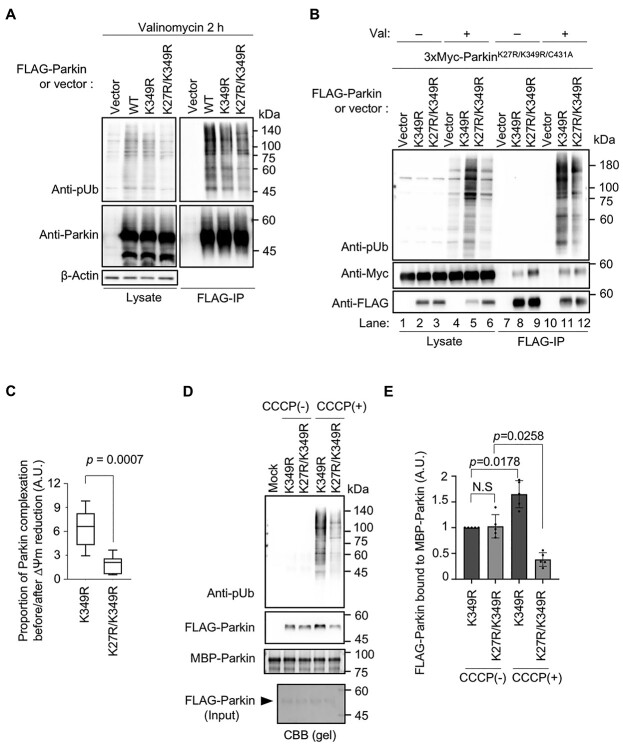
Ubiquitination of the Ubl domain at residue K27 promotes human Parkin complexation upon PINK1 activation. (**A**) K349 in Parkin is one of the autoubiquitination sites. HEK293T cells transfected with FLAG-Parkin or a control vector were treated with 10 μm valinomycin for 2 h. Next, cells were subjected to immunoprecipitation with anti-FLAG and phospho-ubiquitin (pUb) chains attached to Parkin were detected. (**B**) Parkin self-association before and after valinomycin (Val) treatment. HEK293T cells transfected with FLAG-Parkin or a control vector along with 3×Myc-Parkin K27R/K349R/C431A were treated with (+) or without (−) 20 μm Val for 1 h. Cells were then subjected to immunoprecipitation with anti-FLAG, and the co-precipitated 3×Myc-Parkin K27R/K349R/C431A with FLAG-Parkin K349R or K27R/K349R was detected. Notably, Parkin K27R/K349R reproducibly had a higher affinity than Parkin K349R to 3×Myc-Parkin K27R/K349R/C431A under steady-state conditions [i.e. Val (−)] by an unknown reason. (**C**) Formation of the Parkin complex is impaired by K27R mutation. The proportion of newly formed Parkin complex after reduction in mitochondrial membrane potential (ΔΨm) is shown as the increase in 3×Myc-Parkin K27R/K349R/C431A signals normalized to FLAG-Parkin signals after valinomycin treatment (*n* = 8 independent experiments, two-tailed Student’s *t*-test). The ratios were calculated from the experiment shown in (B). A.U., arbitrary unit. (**D**) Formation of the Parkin complex is impaired by K27R mutation *in vitro*. FLAG-Parkin purified from HEK293T cells treated with or without CCCP was subjected to *in vitro* pull-down assay with MBP-Parkin. Phospho-ubiquitination of FLAG-Parkin that bound to MBP-Parkin was also confirmed with anti-pUb antibody. (**E**) Quantification of FLAG-Parkin bound to MBP-Parkin. The intensity of co-precipitated FLAG-Parkin was normalized with each MBP-Parkin, and the value of Parkin K349R without CCCP was set to 1. One-way ANOVA with Tukey–Kramer test (*n* = 5 independent experiments).

The ubiquitin attached to the K27 residue in the Ubl domain is likely to be phosphorylated by PINK1, which could contribute to the mitochondrial tethering and activation of the remaining latent Parkin. To test this possibility, we assessed the formation of the human Parkin complex before and after PINK1 activation in human cultured cells, and *in vitro*. Our mass spectrometry analysis showed that residues K27, K48 and K349 were ubiquitinated upon Parkin activation (Supplementary Material, Fig. S1). Moreover, ubiquitination of Parkin at residue K349 has also been observed in previous studies (27,28). To reduce ubiquitin self-modification, we employed Parkin K349R and Parkin K27R/K349R as probes and Parkin K27R/K349R/C431A as the self-association partner with reduced autoubiquitination (Fig. 6A and B). When PINK1 was active, which would lead to the ubiquitination and phosphorylation in the Ubl domain at K27, the formation of phospho-polyubiquitin in the cells was reduced by K27R (Lane 6 versus Lane 5 in Fig. 6B). In this setting, the binding of Parkin K27R/K349R/C431A to Parkin K349R increased by 6.5-fold on average (anti-Myc, Lane 11 versus Lane 8 in Fig. 6B and C). In contrast, the increase in its binding to Parkin K27R/K349R was approximately 1.8-fold (anti-Myc, Lane 12 versus Lane 9 in Fig. 6B and C). Because Parkin is subjected to degradation upon activation in cells, it is difficult to compare the self-binding ability of Parkin K349R and Parkin K27R/K349R, which have different activation efficiencies *in vivo*, even though it is possible to compare the self-binding ability of these Parkin variants before and after activation. To overcome this problem, we performed an *in vitro* pull-down assay using recombinant maltose-binding protein (MBP)-Parkin and FLAG-Parkin purified from cultured cells treated with or without mitochondrial uncoupler carbonyl cyanide m-chlorophenyl hydrazone (CCCP). CCCP-activated Parkin K349R bound more to MBP-Parkin than to CCCP-activated Parkin K27R/K349R (Fig. 6D and E). These results suggested that Parkin formed a complex probably through the phospho-ubiquitin adduct at site K27.

**Figure 7:**
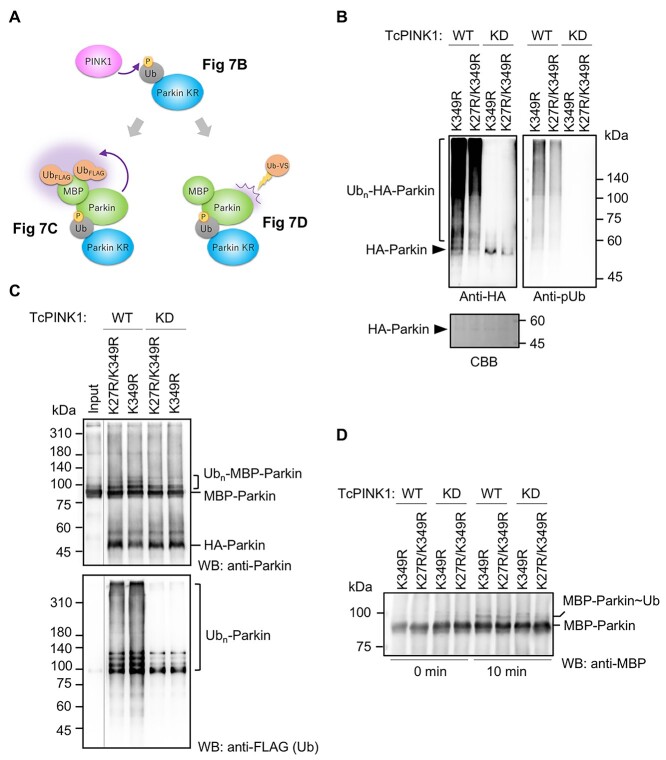
Phospho-ubiquitinated human Parkin activates Parkin *in trans*. (**A**) Overview of experiments to detect *in trans* activation of Parkin. (**B**) Preparation of phospho-ubiquitinated Parkin. Recombinant HA-Parkin purified from bacteria was subjected to the ubiquitination assay in the presence of recombinant TcPINK1 WT or KD. Phospho-ubiquitination of Parkin was confirmed by western blot using anti-pUb. (**C**) MBP-Parkin ubiquitination assay using FLAG-ubiquitin, UBE1 and UbcH7 in the presence of HA-Parkin prepared as in (B). Input; MBP-Parkin without HA-Parkin. (**D**) Detection of Parkin self-activation by Ub-VS in the presence of phospho-ubiquitinated Parkin. MBP-Parkin was incubated with HA-Parkin [prepared as in (B)] for 10 min. MBP-Parkin~Ub; MBP-Parkin with Ub-VS covalently bound to its catalytic cysteine. Two replications were performed for each experiment in (C) and (D), and the representative results are shown.

*In vitro* E3 assays were also performed using recombinant HA-Parkin K349R and K27R/K349R that were treated with *Tribolium castaneum* PINK1 (TcPINK1) WT or kinase-dead (KD). We evaluated the self-ubiquitination and conformational change of WT MBP-Parkin incubated with HA-Parkin K349R or K27R/K349R as depicted in Fig. 7A. We confirmed by mass spectrometry that recombinant HA-Parkin purified from *Escherichia coli* was subject to K27 ubiquitination, as was in cultured cells (Fig. 7B and Supplementary Material, Fig. S3A). Phosphorylation of ubiquitin chains was confirmed by western blotting and mass spectrometry, suggesting that a certain percentage of K27 ubiquitin adduct was phosphorylated (Fig. 7B and Supplementary Material, Fig. S3B). The *in vitro* ubiquitination assay indicated that TcPINK1-activated HA-Parkin K349R enhanced the MBP-Parkin self-ubiquitination more than TcPINK1-activated HA-Parkin K27R/K349R (Fig. 7C). To further confirm that the ubiquitination is a consequence of MBP-Parkin activation after its conformational change (but not ubiquitination by HA-Parkin), ubiquitin-vinyl sulfone (Ub-VS) was used to estimate the exposure of the MBP-Parkin catalytic site. The Ub-VS assay suggested that PINK1-activated HA-Parkin stimulated the conformational change of MBP-Parkin into the activated state (Fig. 7D). However, we could not detect an apparent difference between PINK1-activated HA-Parkin K349R and HA-Parkin K27R/K349R, probably due to the rapid reaction of Ub-VS or the contribution of phospho-ubiquitin adduct(s) of HA-Parkin other than K27 (Fig. 7D).

**Figure 8:**
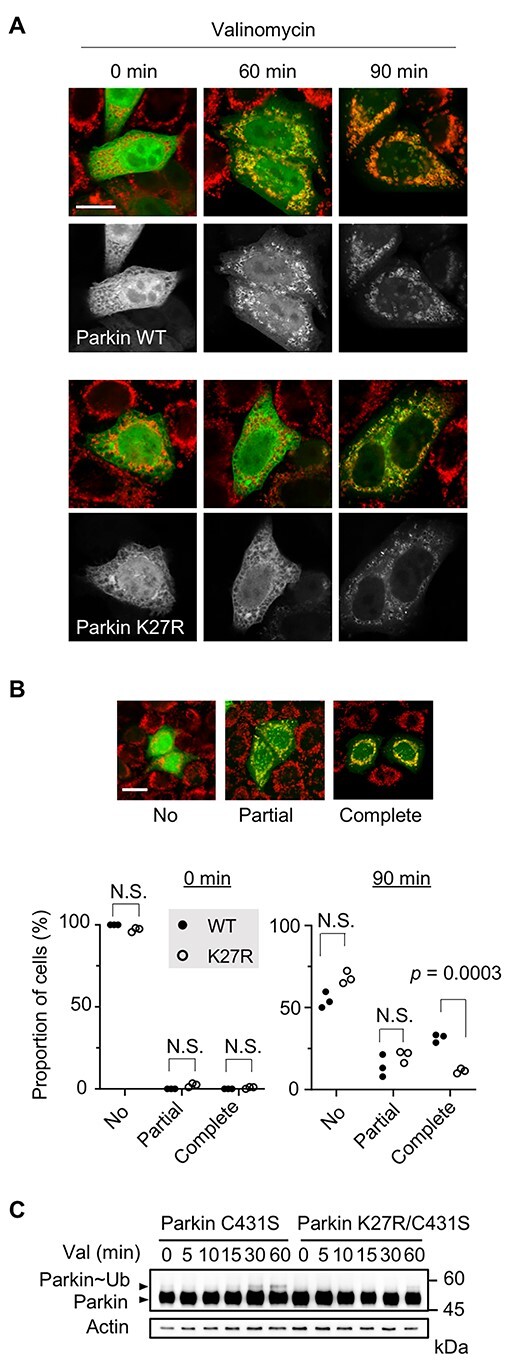
Ubiquitination-defective K27R mutation delays human Parkin activation. (**A**) Delay of mitochondrial translocation of human Parkin by K27R. HeLa cells transfected with EGFP-Parkin WT or K27R (green) were treated with 20 μm valinomycin for the indicated times. Mitochondria (red) were visualized with anti-TOM20 immunostaining. Single channel images of EGFP-Parkin (gray scale) were also shown. Scale bar = 15 μm. (**B**) Mitochondrial translocation efficiency of Parkin WT and K27R. HeLa cells expressing GFP-Parkin were treated as in (A). Completely overlapped, partially overlapped (Partial) or non-overlapped (No) cells with the TOM20 signal were counted (*n* = 3 independent experiments and each value is the mean of >200 cells. Two-tailed Student’s *t*-test). Scale bar = 15 μm. (**C**) Delay in human Parkin activation by K27R mutation. HeLa cells transfected with 3×Myc-Parkin C431S or 3×Myc-Parkin K27R/C431S were treated with 20 μm valinomycin for the indicated time points. Parkin-ubiquitin oxyester species (Parkin~Ub) are detected. Actin served as a loading control.

In another assay using cultured cells, we confirmed that the K27R mutation delayed Parkin mitochondrial translocation when the mitochondrial membrane potential was reduced by valinomycin treatment (Fig. 8A and B). Furthermore, the activation of Parkin E3, by assessing the C431S-oxyester formation with ubiquitin in response to valinomycin treatment (29), was detected within 30 min in the case of Parkin containing an intact Ubl domain, whereas the K27R mutation delayed the activation by more than 30 min (Fig. 8C). These results supported that Parkin ubiquitination at K27 contributed to rapid Parkin activation through Parkin complexation.

## Discussion

In this study, we found that ubiquitin modification at K27 increased Parkin activity, suggestive of a mechanism that promotes Parkin complexation and activation. Replacement of the corresponding K residue with R in *Drosophila* reduced mitochondrial fragmentation, mitochondrial motility arrest and cytotoxicity by PINK1 and Parkin co-expression.

Previously, a Parkin complementation assay revealed Parkin complexation using fluorescence correlation spectroscopy (29). However, the components of the complex remain unclear. The Parkin complementation assay, in which E3-dead Parkin is recruited to the mitochondria in the presence of active PINK1 and Parkin with residual E3 activity, can be interpreted by the binding of Parkin to phosphorylated ubiquitin chains generated on the mitochondria.

**Figure 9:**
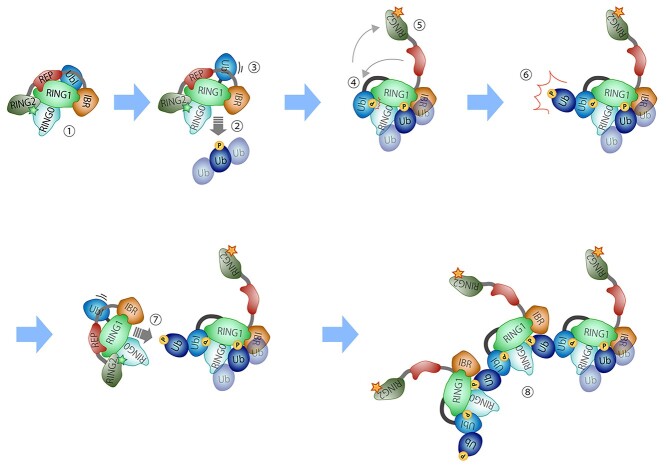
A model for the complexation and activation of Parkin via the Ubl domain ubiquitination. When latent Parkin (1) binds to phosphorylated ubiquitin (2), Ubl is released from the RING1 domain (3). The released Ubl is phosphorylated by PINK1 and binds to the RING0 domain (4), and the RING2 domain is simultaneously released from the RING0 domain, activating ubiquitin ligase (E3) activity (5). E3 causes ubiquitination of the Ubl domain at residue K27 and subsequent phosphorylation of the ubiquitin at residue S65 by PINK1 (6). A part of the latent Parkin binds to the phosphorylated ubiquitin on the Ubl domain, forming a complex and activating E3 (7). This reaction occurs in succession to form the Parkin activation complex (8).

As a suggestion for the Parkin complex from structural analysis, Kumar *et al.* (30) suggested that a cryptic ubiquitin-binding site may be created between the Ubl and IBR domains with the binding of phospho-ubiquitin. However, in an activation model proposed by Sauve *et al.* and Gladkova *et al.* (4,5), the phosphorylated Ubl domain moves more dynamically and is anchored to the RING0 domain. In this model, complexation is not required (5). Our model is based on Sauvé/Gladkova’s model, but also suggests Parkin complexation; that is, our model involves recruitment of Parkin to phosphorylated ubiquitin attached at K27 in the Ubl domain and subsequent activation of Parkin (Fig. 9). Although it is difficult to test this concept *in vitro* owing to a lack of appropriate technology for controlling phosphorylated ubiquitin modifications precisely, the *Drosophila* phenotypic assay supported this concept.

A previous study suggests that mono-ubiquitination is predominant when Parkin is self-ubiquitinated (31). This observation suggests that K27 is likely to be modified by phosphorylated mono-ubiquitin. Why the phospho-ubiquitin modification of K27 contributes more to activation than that of other sites (e.g. K48) is an open question; K27 may be located at a site that is easily accessible to the latent form of Parkin. Recently, an alternative mechanism of Parkin activation has been proposed (32). In this model, phospho-ubiquitin binds to the RING0 of Parkin instead of phospho-Ubl. Our *in trans* activation model is compatible with this model because the latent Parkin is activated via the phospho-ubiquitinated Parkin although our results of fly phenotypes and *in vitro* experiments suggest that phospho-ubiquitination at K27 is an adjunct to the feedforward activation mechanism of Parkin.

Intriguingly, phenotypic differences existed between flies harboring the K56R and K56N mutations; dParkin K56N, similar to dParkin S94A, resulted in a complete loss-of-function phenotype. In contrast, dParkin K56R resulted in a milder phenotype than dParkin K56N. Considering that the activity of dParkin K77R was almost unchanged compared with that of WT dParkin, ubiquitin addition at residue K56 (K27 in human) in the Ubl domain appeared to be physiologically important in the process of Parkin activation. We also point out the possibility that the K56N mutation destabilizes dParkin. We created 10 independent lines of dParkin K56N transgenics. However, none of them exhibited equal or higher expression than WT dParkin. This fact may indicate that the K56N mutation destabilizes dParkin, which may contribute to the loss-of-function phenotype.

Some studies have described differences in molecular behavior between human Parkin K27N and WT Parkin (17,23), while others could not detect any differences between Parkin K27N/R and WT Parkin (20,22). This discrepancy could be attributed to issues with the expression levels of these proteins or the difference between conditions *in vivo* and *in vitro*. For example, our previous study demonstrated only a small delay in mitochondrial translocation of phospho-resistant Parkin S65A (15). However, we later realized that this was due to the use of cells ectopically expressing PINK1. Through endogenous PINK1 expression, we then detected a significant delay in mitophagy, which is further reinforced in our *Drosophila* study (12). Therefore, in the current study, we exercised caution to avoid Parkin overexpression in the cultured cell experiments.

The K27 residue in the Ubl domain is located at the first α-helix in the Ubl fold. The crystal structure of Parkin suggests that the side chain of K27 forms hydrogen bonds with the side chains of A38 and L41 in the loop region between the first α-helix and the third β-sheet and with that of N52 in the loop region between the fourth β-sheet and the second α-helix, thereby maintaining the Ubl fold (Supplementary Material, Fig. S4) (33–35). Accordingly, the Ubl domain of Parkin K27N is suggested to be unstable (36) and remains unreleased from the Parkin RING1 domain even after PINK1 activation (23). Supporting this notion, the efficiency of phosphorylation of Parkin K27N by PINK1 is lower than that of WT Parkin (14,17). Thus, the K27N mutation resulted in a Parkin protein not only resistant to ubiquitination, but also with impaired dynamic movement of the Ubl domain.

The two salt bridges found in ubiquitin (K11–E34 and K27–D52) are not conserved in the Ubl domain (37). A ubiquitin mutant containing K27M results in disruption of the K27–D52 salt bridge, which leads to ubiquitin tending to exhibit a stable unfolding transition state and, therefore, being kinetically unstable (37). In contrast, the salt bridge-free Ubl domain is suggested to have a more flexible structure, thereby supporting the elaborate Parkin activation mechanism. Ubiquitin modification of the Ubl domain K27 residue along with phosphorylation of S65 in both the Ubl domain and ubiquitin could lead to dynamic changes in the structure of the Ubl domain and help activate Parkin in *cis* and *trans* (35).

In conclusion, our study using *Drosophila* genetics and human cultured cells suggested that ubiquitination of Parkin at K27 (K56 in *Drosophila*) may contribute to the formation of the Parkin activation complex and facilitate rapid Parkin activation. Although this potential mechanism should be tested in additional studies in the future, a detailed understanding of the Parkin activation mechanism will be useful for the development of compounds targeting Parkin.

## Materials and Methods

### *Drosophila* stock preparation

Fly culture and crosses were performed using standard fly food containing yeast, cornmeal and molasses, and the flies were raised at 25°C unless otherwise indicated. Complementary DNAs for dParkin K56N, K56R, K77R, K56R/K77R, C449A, K231N, K296W and ∆Ubl (amino acids 107–482) synthesized by GenScript (Tokyo, Japan) or generated by PCR-based mutagenesis were subcloned into the pUAST vector. *UAS-dParkin K56N*, *K56R*, *K77R*, *K56R/K77R*, *C449A*, *K231N*, *K296W* and *∆Ubl* transgenic lines were generated on the *w−* background (BestGene Inc., Chino Hills, CA, USA). Fly stock expressing the *Park^25^* null allele, a kind gift from Dr Pallanck, was prepared as described previously (38). All other fly stocks and GAL4 lines used in this study were obtained from the Bloomington Drosophila Stock Center (Bloomington, IN, USA) and have been previously described: *Park^1^* (39), *Park^Δ21^* (40), *UAS-PINK1* (41), *UAS-dParkin WT* (12), *UAS-dParkin S94A* (12) and *UAS-dParkin S94E* (12). *Park^1^*/*Park^Δ21^* and *Park^25^*/*Park^25^* were used as *dParkin*-deficient alleles. *PINK^15^* (Bloomington #51649) was used as a *PINK1*-deficient allele.

### Plasmid DNA construction

pcDNA3-3×Myc-tagged human Parkin C431S was prepared as described previously (15). pcDNA3-FLAG-tagged human Parkin K27R and human Parkin K27R/K349R were generated using pcDNA3-FLAG-tagged human Parkin (42) as the template for PCR-based mutagenesis. pcDNA3-3×Myc-tagged human Parkin K27R/K349R/C431A and K27R/C431S were generated using pcDNA3-3×Myc-tagged human Parkin (15) as the template for PCR-based mutagenesis. The sequences of all genes were validated by DNA sequencing. For enhanced green fluorescent protein (EGFP)-Parkin, human Parkin cDNA was cloned into the pEGFP vector and the K27R mutation was introduced by PCR-based mutagenesis. The primer sequences used for mutagenesis were as follows: 5′-GAGAGGTGGTTGCTAAGCGACAG-3′ (Parkin K27R forward), 5′-TGAGCTGGAAGATGCTGGTGTC-3′ (Parkin K27R reverse); 5′-TGACCAGAGGAGAGTCACCTGCG-3′ (Parkin K349R forward), 5′-CGCAGGTGACTCTCCTCTGGTCA-3′ (Parkin K349R reverse); 5′-AAAATGGAGGCGCCATGCACATGA-3′ (Parkin C431A forward), 5′-TCATGTGCATGGCGCCTCCATTTT-3′ (Parkin C431A reverse) and 5′-AAAATGGAGGCTCCATGCACATGA-3′ (Parkin C431S forward), 5′-TCATGTGCATGGAGCCTCCATTTT-3′ (Parkin C431S reverse).

### Western blot analysis and immunoprecipitation

HEK293 cells (RIKEN BRC, Wako, Japan) were cultured in Dulbecco’s modified Eagle medium (cat. no. 0845816; Nacalai Tesque, Kyoto, Japan) supplemented with 10% fetal bovine serum (Gibco, Thermo Fisher Scientific, Waltham, MA, USA) and 1% penicillin–streptomycin. The cells were transfected with the expression plasmids using PEI Max reagent (cat. no. 24765; Polysciences, Warrington, PA, USA). After 24 h, the cells were treated with 10 μm valinomycin (cat. no. 36136-14; Nacalai Tesque) for 2 h and then lysed in lysis buffer containing 50 mm Tris–HCl (pH 7.4), 120 mm NaCl, 5 mm EDTA, 1% Triton X-100 and 10% glycerol supplemented with a protease inhibitor cocktail (cat. no. 03969-21; Nacalai Tesque) and phosphatase inhibitor cocktail (cat. no. 07575-51; Nacalai Tesque). Cell lysates were centrifuged at 20 000*g* for 10 min at 4°C. Ten microliters of the supernatant was retained as the input for immunoprecipitation. The rest of the supernatant was mixed with 10 μl anti-FLAG M2 affinity gel (cat. no. A2220; Sigma-Aldrich, Merck, Darmstadt, Germany), rotated at 4°C for 3 h, spun down at 1000*g* for 1 min and washed with the lysis buffer three times for 10 min each. Proteins were eluted for 1 min at 22°C with 10 μl 3× sodium dodecyl sulfate (SDS) sample buffer (188 mm Tris–HCl, 3% SDS, 30% glycerol, 0.01% bromophenol blue, 15% β-mercaptoethanol). The fly brain and thorax tissues were directly homogenized in 20 μl of 3× SDS sample buffer using a motor-driven pestle. The same amounts of protein were subjected to SDS-polyacrylamide gel electrophoresis, and the separated proteins were transferred onto polyvinylidene fluoride membranes. The membranes were blocked for 1 h at 22°C with 5% milk in phosphate-buffered saline (PBS) containing 0.05% Tween20 (PBST) and then incubated overnight with primary antibodies at 4°C. After washing three times with PBST, the membranes were incubated with horseradish peroxidase-conjugated secondary antibodies at 22°C for 1 h. After washing three times with PBST, signals were detected with Immobilon Forte Western HRP substrate (cat. no. WBLUF0500; Millipore, Merck, Darmstadt, Germany). The blotting images were obtained using Fusion FX6 Edge (Vilber-Lourmat, Marne-la-Vallée, France). Band intensities were quantified using Evolution Capt software ver. 18.06 (Vilber-Lourmat).

Rabbit anti-dParkin polyclonal antibody was raised against recombinant MBP-tagged dParkin (275–482 amino acids) produced in the *E. coli* strain Rosetta 2 (Novagen, Merck, Darmstadt, Germany) (12). Rabbit anti-*Drosophila* Mitofusin polyclonal antibody was raised against a mixture of synthetic peptides (C-DTVDKSGPGSPLSRF and CIQNELDIFEHNYISPQ) and purified through affinity chromatography (Japan Bio Services, Asaka, Japan) (15). The following primary antibodies were used for western blotting: anti-dParkin (1:1000, in-house), anti-Mitofusin (1:500, in-house), anti-NDUFS3 (1:1000; cat. no. ab14711; Abcam, Cambridge, UK), anti-ATP5A (1:5000; cat. no. 15H4C4; Abcam), anti-actin (1:10 000; cat. no. MAB1501; Millipore), anti-FLAG (1:1000 for cells, 1:10 000 for *in vitro* ubiquitination assay; cat. no. F1804; Sigma-Aldrich), anti-Myc (1:1000; cat. no. 05–724; Sigma-Aldrich), anti-HA (1:10 000; cat. no. 901501; BioLegend, San Diego, CA, USA), anti-MBP (1:30 000; cat. no. E8030S; New England Biolabs, Ipswich, MA, USA), anti-phospho-ubiquitin (1:1000; cat. no. 62802; Cell Signaling Technology, Danvers, MA, USA) and anti-human Parkin (1:1000; cat. no. 4211; Cell Signaling Technology).

### Immunofluorescence and confocal microscopy

Fly tissues were dissected in PBS and fixed in 4% paraformaldehyde for 50 min (for the thorax) or 30 min (for the brain) at 22°C. Anti-tyrosine hydroxylase (1:1000; cat. no. MAB318; Millipore) and anti-PDF (1:500; cat. no. PDF C7; Developmental Studies Hybridoma Bank, IA, USA) were used to visualize dopaminergic neurons and PDF neurons, respectively. The mitochondrial morphology was analyzed by whole-mount immunostaining, as described previously (43). Stacks of mitochondrial images were acquired at 0.35 μm intervals using a 63× oil immersion objective under a Leica SP5 confocal microscope system (Leica Microsystems, Wetzlar, Germany). Images were reconstructed using a series of stacked images with ImageJ Z-projection tool (National Institutes of Health, Bethesda, MD, USA). Mitochondrial number and length were assessed using the Analyze Particles function in ImageJ software.

For the experiment using mammalian cultured cells, HeLa cells (RIKEN BRC) were cultured in Dulbecco’s modified Eagle medium, supplemented with HEPES without l-glutamine and sodium pyruvate (cat. no. 11585-75; Nacalai Tesque) but with 10% fetal bovine serum (Gibco) and 1% penicillin–streptomycin, using Millicell EZ slides (cat. no. PEZGS0816; Millipore). The cells were transfected with plasmids expressing EGFP-tagged Parkin using Lipofectamine 2000 Transfection Reagent (cat. no. 11668019; Invitrogen, Thermo Fisher Scientific) following the manufacturer’s instructions. Cells were treated with 10 μm valinomycin (cat. no. 36136-14; Nacalai Tesque) before fixation. Cells were washed with PBS, fixed in 4% paraformaldehyde in PBS for 10 min at 22°C, washed three times with PBS, permeabilized with 0.05% digitonin (cat. no. 048-02124; FUJIFILM Wako, Osaka, Japan) in PBS for 15 min and blocked with 0.1% gelatin in PBS for 30 min. Cells were then incubated for 1 h at 22°C with anti-TOM20 (1:1000; cat. no. 612278; BD Biosciences, Franklin Lakes, NJ, USA) in 0.1% gelatin in PBS, followed by incubation with Alexa Fluor 594-AffiniPure goat anti-rabbit IgG (1:1000; cat. no. 111-585-045; Jackson Laboratory, Bar Harbor, ME, USA) in 0.1% gelatin in PBS for 1 h. Finally, the cells were mounted with Fluoro-KEEPER antifade reagent (cat. no. 12593-64; Nacalai Tesque). Images were obtained using a Leica SP5 confocal microscope with a 63× oil immersion objective and processed with ImageJ 1.52 software (National Institutes of Health).

### *In vitro* Parkin activation assay

In this assay, pMal-TcPINK1 (WT and KD D359A), pET15b-SUMO-HA-human Parkin (provided by Dr Muqit) and pMal-human Parkin (11) were used as bacterial expression plasmids for recombinant protein production. MBP fusion-*T. castaneum* PINK1 (TcPINK1) was purified from *E. coli* BL21/PG-KJE8 (cat. no. 9121; Takara Bio, Kusatsu, Japan). His_6_-SUMO-HA-Parkin and MBP-human Parkin were purified from *E. coli* and BL21-CodonPlus(DE3)-RIL (cat. no. 230245; Agilent, Santa Clara, CA, USA). Recombinant human ubiquitin activating enzyme (UBE1; E-305-025), human UbcH7 (E2-640-100) and human His_6_-SENP1 (E-700-050) were purchased from R&D Systems (Minneapolis, MN, USA). Bovine ubiquitin and FLAG-ubiquitin were purchased from Sigma-Aldrich.

His_6_-SUMO-HA-Parkin (6.4 μm) bound to Ni-NTA beads was treated with 1.4 μm TcPINK1, 0.1 μm UBE1, 3 μm UbcH7 and 22 μm ubiquitin in a kination/ubiquitination buffer [50 mm Tris, pH 8.2, 120 mm NaCl, 5 mm MgCl_2_, 1 mm CaCl_2_, 0.2 mm dithiothreitol (DTT) and 4 mm ATP] at 30°C for 120 min to generate phospho-ubiquitinated Parkin. Ubiquitination of Parkin at K27 and phosphorylation of ubiquitin were confirmed by mass spectrometry (Supplementary Material, Fig. S3). The Parkin-bound beads were washed with SENP1 cleavage buffer (50 mm Tris, pH 8.2, 200 mm NaCl, 10% glycerol, 0.5 mm TCEP and 1 mm DTT) and treated with His_6_-SENP1 at 30°C for 120 min to release phospho-ubiquitinated HA-Parkin from Ni-NTA beads. Phospho-ubiquitinated HA-Parkin solution was passed through an amylose resin column to remove MBP-TcPINK1 that may have been included in the HA-Parkin fraction. Resultant phospho-ubiquitinated HA-Parkin was subjected to a ubiquitination assay using MBP-Parkin. The reaction was performed in a ubiquitination buffer (50 mm Tris, pH 8.2, 120 mm NaCl, 5 mm MgCl_2_, 0.1 mm DTT, 4 mm ATP) containing 0.2 μm UBE1, 3 μm UbcH7, 21 μm FLAG-ubiquitin and 0.4 μm MBP-Parkin, with or without 0.6 μm phospho-ubiquitinated HA-Parkin. The ubiquitination reaction was performed at 30°C for 60 min.

### Ub-VS assay

Recombinant phospho-ubiquitinated HA-Parkin was prepared as described above. An amount of 2 μm Ub-VS (R&D, E-202) and 0.2 μm MBP-Parkin and 0.6 μm HA-Parkin were incubated in Ub-VS assay buffer (25 mm Tris, pH 8.5, 120 mm NaCl, 2 mm TCEP) at 30°C for 10 min.

### *In vitro* parkin pull-down assay

HEK293 cells were transiently transfected with FLAG-Parkin K349R and FLAG-Parkin K27/K349R and were treated with or without 20 μm CCCP for 1 h. FLAG-Parkin K349R and FLAG-Parkin K27/K349R were immunopurified from the HEK293 cell lysate using anti-FLAG M2 affinity gel and eluted with 400 μg/ml 3× FLAG peptide. The amount of FLAG-Parkin obtained was determined by Coomassie Brilliant Blue staining and following comparison to a known concentration of BSA. Phospho-ubiquitinated FLAG-Parkin (0.25 μm) was incubated with 0.35 μm MBP-Parkin that bound to amylose resin in a binding buffer (0.5% Triton X-100, 50 mm Tris, pH 8.2, 150 mm NaCl, 0.5 mg/ml BSA and 0.5 mm TCEP) for 60 min at 22°C. After washing the beads with 200 μl wash buffer (0.5% Triton X-100, 50 mm Tris, pH 8.2, 200 mm NaCl and 0.5 mm TCEP) three times, FLAG-Parkin co-precipitated with MBP-Parkin was analyzed with anti-FLAG antibody and anti-pUb antibody.

### Liquid chromatograph-mass spectrometry analysis

*PINK1^−/−^* mouse embryonic fibroblasts expressing HA-Parkin and PINK1-FLAG were treated with or without 30 μm CCCP for 30 min. HA-Parkin immunopurified with anti-HA-conjugated agarose beads (cat. no. 014-23 081; FUJIFILM Wako) was reduced with 10 mm DTT for 30 min, alkylated with 50 mm iodoacetamide for 30 min in the dark and digested with trypsin (11). Liquid chromatograph-mass spectrometry (LC–MS/MS) analysis was performed with a TripleTOF5600 (SCIEX, Framingham, MA, USA) connected to an Ultimate 3000 pump (Thermo Fisher Scientific) and an HTC-PAL autosampler (CTC Analytics, Zwingen, Switzerland). Peptides were separated by a self-pulled needle column (150 mm length, 100 μm ID, 6 μm needle opening) packed with Reprosil-Pur 120 C18-AQ 3 μm reversed-phase material (Dr Maisch, Ammerbuch-Entringen, Germany), using a 65 min gradient of 5–40% B (mobile phase A: 0.5% acetic acid, mobile phase B: 0.5% acetic acid/80% acetonitrile) at a flow rate of 500 nl/min. The MS scan range was *m/z* 300–1500. The top 10 precursor ions were selected in each MS scan for subsequent MS/MS scans. MS scans were performed for 0.25 s, and subsequently 10 MS/MS scans were performed for 0.1 s each.

For the analysis of recombinant proteins, the sample was reduced, alkylated and digested with trypsin as described above. An Orbitrap Fusion Lumos (Thermo Fisher Scientific) connected to an Ultimate 3000 pump and an HTC-PAL autosampler was employed for the analysis. The chromatographic conditions were as described above. The MS scan range was *m/z* 300–1500, and the MS1 orbitrap resolution was 120 000. MS2 spectra were acquired in the data-dependent acquisition mode with the following settings: orbitrap resolution of 15 000, quadrupole isolation window of 1.6, AGC target value of 50 000 and HCD collision energy of 30%.

For peptide identification, the peak list in Mascot generic format was generated using MS Converter version 1.3.0.219 (for TripleTOF 5600) or MaxQuant version 1.6.17.0 (44) (for Orbitrap Fusion Lumos). Peptides and proteins were identified by means of an automated database search using Mascot version 2.7.0 (Matrix Science, Boston, MA, USA) against the human database from UniprotKB/SwissProt release 2019/10 (20 380 sequences) with a precursor mass tolerance of 20 ppm (TripleTOF 5600) or 10 ppm (Orbitrap Fusion Lumos), a fragment ion mass tolerance of 0.1 Da (TripleTOF 5600) or 20 ppm (Orbitrap Fusion Lumos) and strict trypsin specificity allowing for up to two missed cleavages. Carbamidomethyl (C) was set as a fixed modification. Phosphorylation (STY) and GG (K) were set as variable modifications. Peptides were accepted if the Mascot score was over the 95% confidence limit (*P* < 0.05), based on the identity score of each peptide.

### Statistical analysis

Sample size estimation was calculated using GraphPad Prism 6.0 (GraphPad Software Inc., San Diego, CA, USA) for a confidence level of 95%, *α* = 0.05. GraphPad Prism 6.0 (GraphPad Software Inc.) was also used to perform statistical analyses. For comparisons between two specific or multiple groups with normal distribution, two-tailed Student’s *t*-tests and two-way factorial analysis of variance (ANOVA) followed by Tukey’s honest significant difference test were used, respectively. Dunnett’s test was used to compare each of the mutant groups with a single control group. For multi-group comparisons not following normal distribution, Welch ANOVA with Steel test was used to compare each of the mutant groups with a single control group. In all cases, significance was noted at *P* < 0.05. Graphs were plotted using GraphPad Prism 6.0 (GraphPad Software Inc.).

## Data availability

The mass spectrometry raw data and analytical files have been deposited to the ProteomeXchange Consortium (http://proteomecentral.proteomexchange.org) via the jPOST partner repository (https://jpostdb.org) (45) with the data set identifier PXD026554.

**ABBREVIATIONS**
CBB: Coomassie Brilliant Blue;
  E2
  ubiquitin-conjugating enzyme;
  E3
  ubiquitin ligase;
  PBST
  phosphate-buffered saline containing 0.05% Tween20;
  PINK1
  PTEN-induced kinase 1;
  Ubl
  ubiquitin-like;
  dParkin
  *Drosophila* Parkin

## Supplementary Material

Liu_K27_Parkin_HMG_Suppl_info_ddac064
