# Supplementary material for: Ubiquitination at the lysine 27 residue of the Parkin ubiquitin-like domain is suggestive of a new mechanism of Parkin activation
Source: Hum Mol Genet. 2022 Mar 21;31(15):2623–38. doi: 10.1093/hmg/ddac064 (PMC9396936; doi:10.1093/hmg/ddac064)
Supplement: Liu_K27_Parkin_HMG_Suppl_info_ddac064 [file liu_k27_parkin_hmg_suppl_info_ddac064.pdf]

Ubiquitination at the lysine 27 residue of the Parkin ubiquitin-like domain is suggestive of a new mechanism of Parkin activation

Jun-Yi Liu, Tsuyoshi Inoshita, Kahori Shiba-Fukushima, Shigeharu Yoshida, Kosuke Ogata, Yasushi Ishihama, Yuzuru Imai, and Nobutaka Hattori

List of materials included:

Supplementary Material, Fig. S1-S4

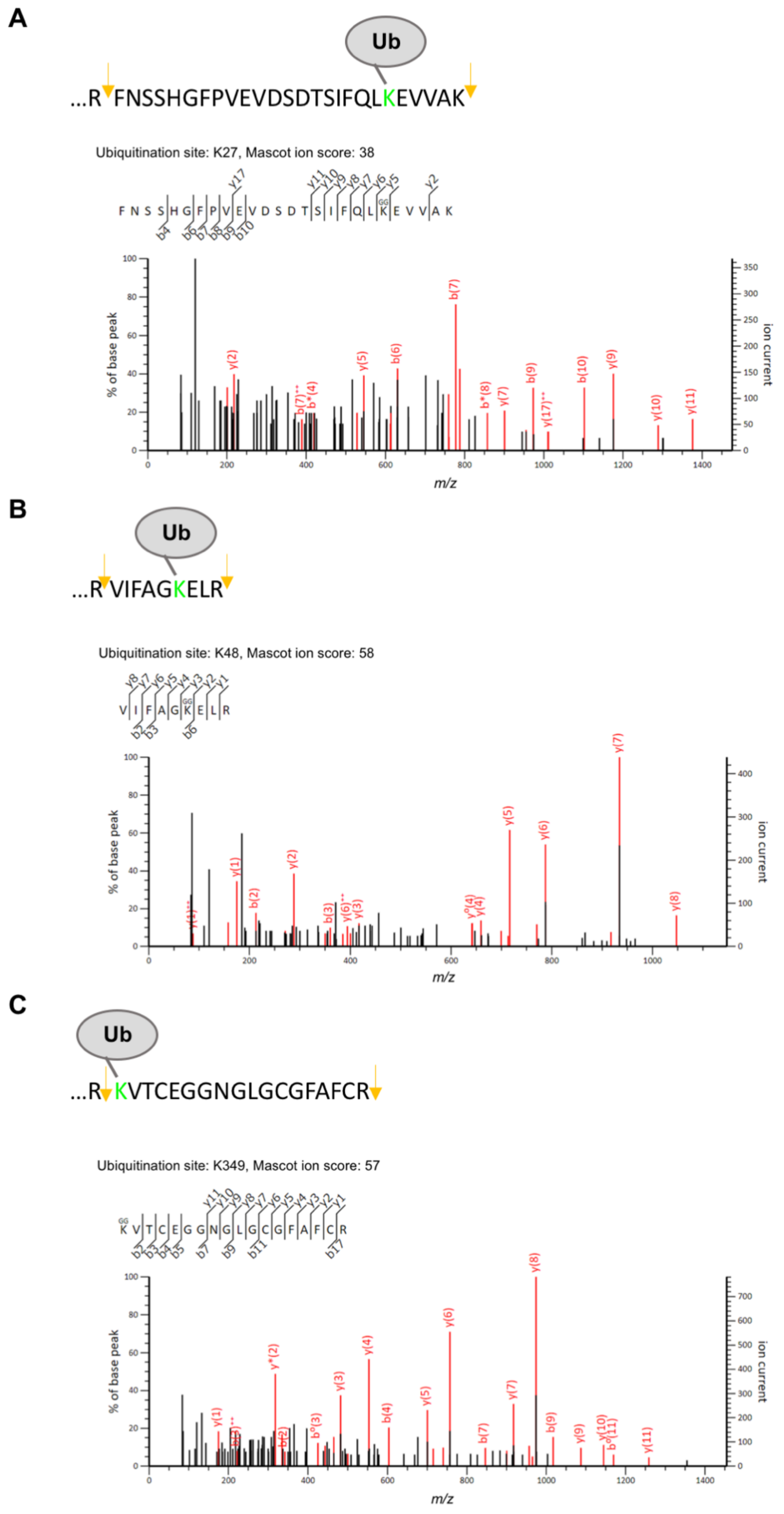
A
Ub
...R FNSSHGFPVEVDSDTSIFQLKEVVAK
Ubiquitination site: K27, Mascot ion score: 38
B
Ub
...R VIFAGKELR
Ubiquitination site: K48, Mascot ion score: 58
C
Ub
...R KVTCEGGNGLGCGFAFCR
Ubiquitination site: K349, Mascot ion score: 57
% of base peak
ion current
m/z

Figure S1. Identification of K27 and K48 ubiquitination in the Parkin Ubl domain. (**A**) *PINK1*$^{-/-}$ mouse embryonic fibroblasts expressing HA-tagged human Parkin and human PINK1-FLAG were treated with or without 30 μM protonophore CCCP for 30 min. HA-Parkin immunopurified with anti-HA-conjugated agarose beads was digested with trypsin and analyzed by nano-scale liquid chromatography-tandem mass spectrometry (LC-MS/MS) (Dionex Ultimate3000 RSLCnano and ABSciex TripleTOF 5600) followed by MASCOT searching (1). An MS/MS spectrum was assigned to FNSSHGFPVEVDSDTSIFQLK*EVVAK (7–32 amino acid (aa) with ubiquitination (Ub) of K27). (**B**) An MS/MS spectrum was assigned to VIFAGK*ELR (43–51 aa with ubiquitination of K48). (**C**) An MS/MS spectrum was assigned to K*VTCEGGNGLGCGFAFCR (349–366 aa with ubiquitination of K349). Yellow arrows indicate the sites cleaved by trypsin.

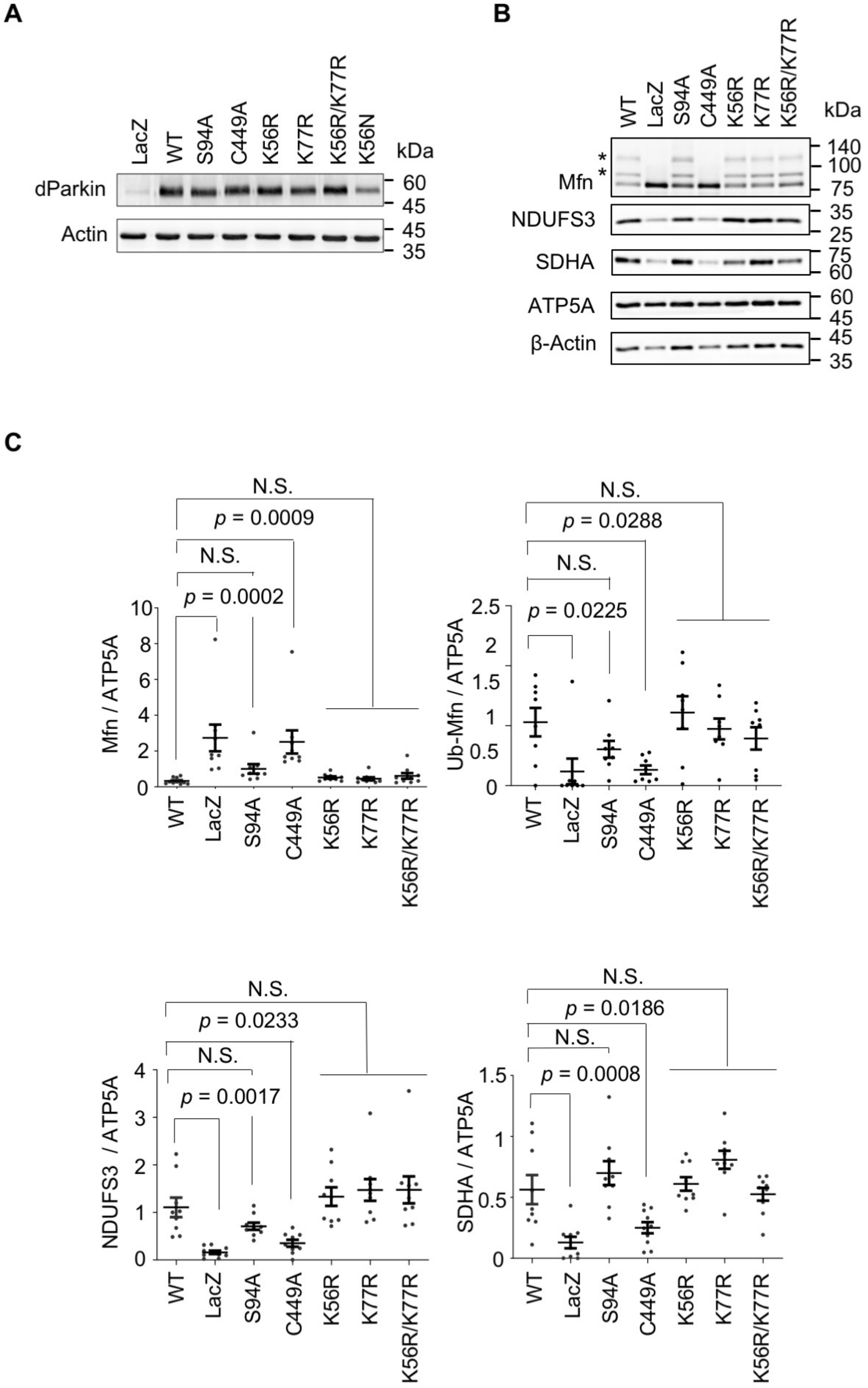
A
LacZ
WT
S94A
C449A
K56R
K77R
K56R/K77R
K56N
kDa
dParkin
60
45
Actin
45
35
B
WT
LacZ
S94A
C449A
K56R
K77R
K56R/K77R
kDa
Mfn
*
*
140
100
75
NDUFS3
35
25
SDHA
75
60
ATP5A
60
45
β-Actin
45
35
C
N.S.
p = 0.0009
N.S.
p = 0.0002
Mfn / ATP5A
N.S.
p = 0.0288
N.S.
p = 0.0225
Ub-Mfn / ATP5A
N.S.
p = 0.0233
N.S.
p = 0.0017
NDUFS3 / ATP5A
N.S.
p = 0.0186
N.S.
p = 0.0008
SDHA / ATP5A
WT
LacZ
S94A
C449A
K56R
K77R
K56R/K77R

Figure S2. Non-ubiquitinated *Drosophila* Parkin mutants have minimal effects on mitochondrial protein levels.

(**A**) Expression of dParkin (WT, S94A, C449A, K56R, K77R, K56R/K77R, and K56N) or β-galactosidase (LacZ) in the *Drosophila* thorax muscle evaluated using the *daughterless-GAL4* driver. LacZ and WT dParkin were used as negative and positive controls, respectively. (**B**) The indicated mitochondrial proteins extracted from the thoraxes of three-day-old adult flies were analyzed by western blotting. ß-Actin and ATP5A were used as loading controls for cytosolic and mitochondrial proteins, respectively. Asterisks indicate putative ubiquitinated Mitofusin (Ub-Mfn). Transgenes were expressed in *dParkin*-deficient flies using the ubiquitous *daughterless-GAL4* driver. (**C**) The band intensities of the indicated mitochondrial proteins were normalized to that of ATP5A. The values represent the mean ± SE from 10 independent samples as in **B**. Comparison was performed using Dunnett's test (*vs.* WT *Drosophila* Parkin). N.S., not significant; NDUFS3, NADH dehydrogenase (ubiquinone) Fe-S protein 3; SDHA, Succinate dehydrogenase complex flavoprotein subunit A; ATP5A, ATP synthase F1 subunit alpha.

A

Parkin K349R, Ubiquitination site: K27, Mascot ion score: 131

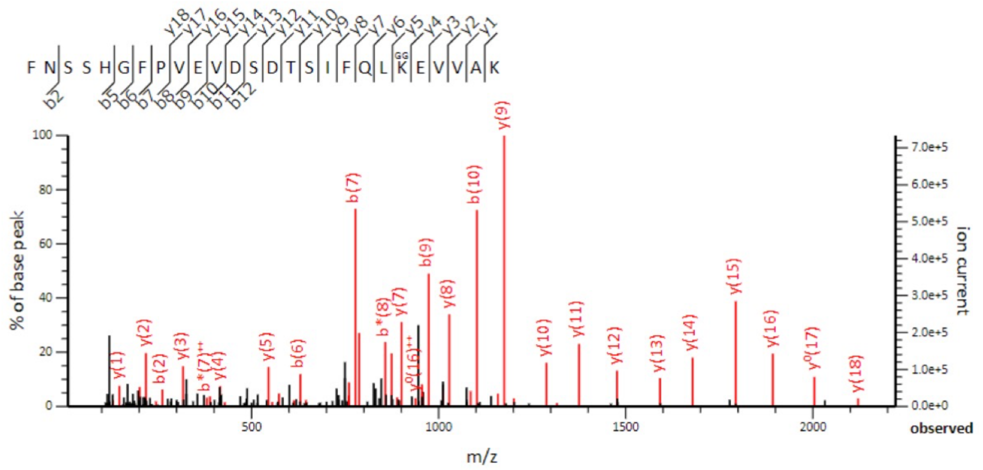

B

Ubiquitin, Phosphorylation site: S65, Mascot ion score: 138

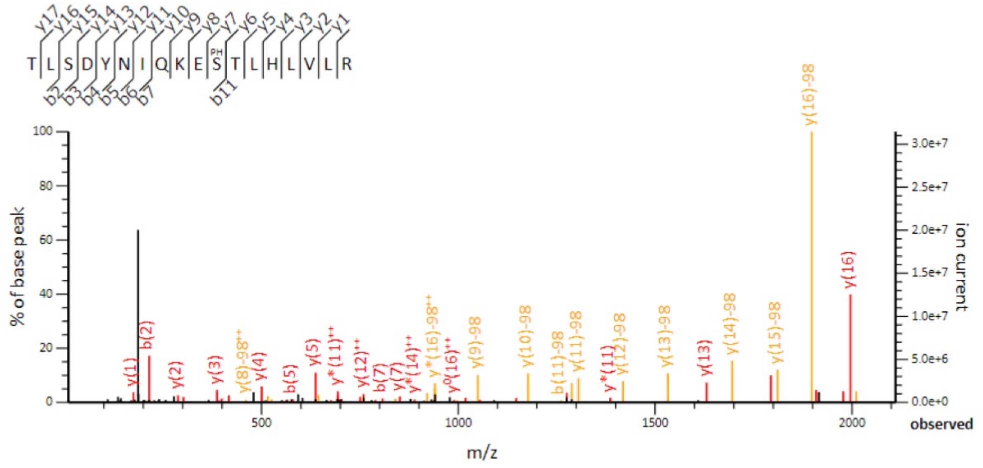

Figure S3. K27 ubiquitination and ubiquitin phosphorylation of recombinant human Parkin.

(A) An MS/MS spectrum was assigned to FNSSHGFPVEVDSDTSIFQLK*EVVAK (7–32 amino acid (aa) with ubiquitination (Ub) of K27). (**B**) An MS/MS spectrum was assigned to TLSDYNIQKES*TLHLVLR (55–72 aa with phosphorylation of S65).

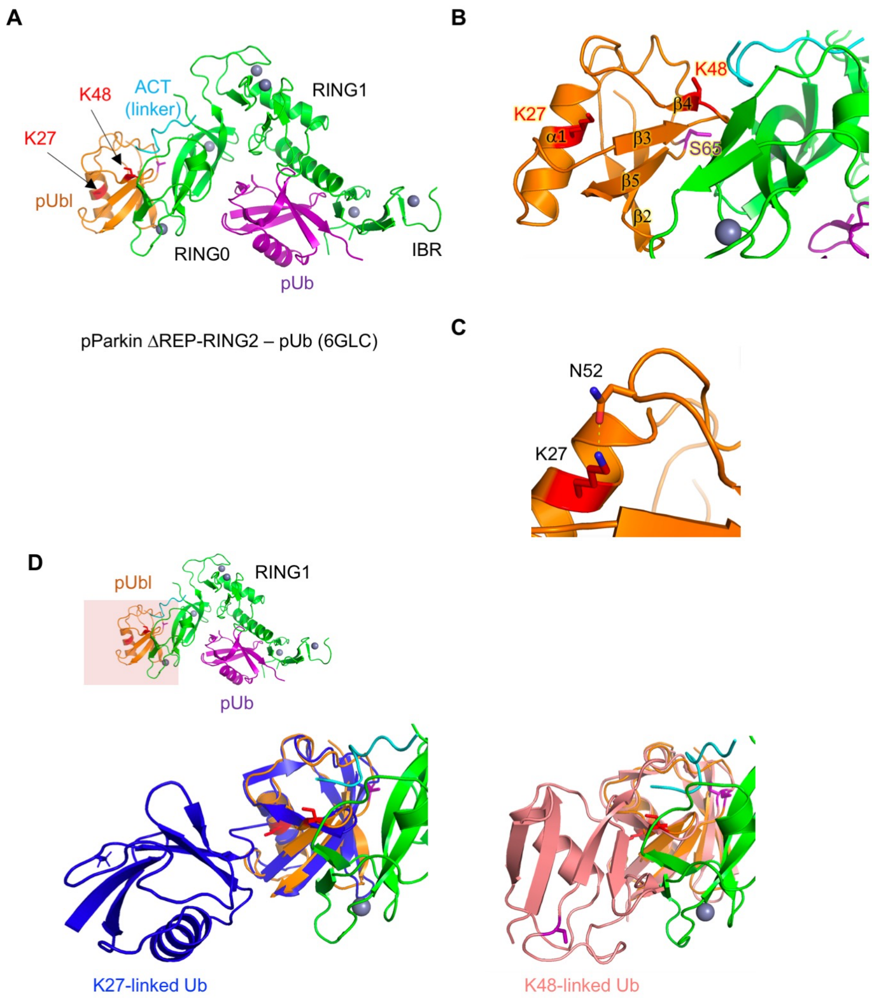

Figure S4. Predicted visual of ubiquitination in the Parkin Ubl domain at residue K27 or K48.

(**A**) The location of K27 and K48 residues in phospho-Ser65 Parkin. Phospho-Ser65 Parkin lacking the REP and RING2 domains, and phospho-Ser65 ubiquitin (pUb) are depicted by the ribbon model. pUbl, phospho-Ser65 Ubl domain. (**B**) Higher magnification of the Ubl domain containing the K27 and K48 residues. The position of S65 is also depicted. (**C**) Hydrogen bonding between K27 and N52 side chains determined by *in silico* prediction. (**D**) Topological prediction of the K27-linked (left) and K48-linked (right) Ub modification in the Parkin Ubl domain by superposition of diUb (blue or pink) onto the Parkin Ubl domain (orange). Crystal structures of Parkin lacking the REP and RING2 domains (PDB ID: 6GLC), K27-linked diUb (PDB ID: 5UJN), and K48-linked diUb (PDB ID: 2AUL) were created using PyMOL (version 2.0.7., Schrödinger, Inc.).

References

1. Shiba-Fukushima, K., Imai, Y., Yoshida, S., Ishihama, Y., Kanao, T., Sato, S., and Hattori, N. (2012) PINK1-mediated phosphorylation of the Parkin ubiquitin-like domain primes mitochondrial translocation of Parkin and regulates mitophagy. *Scientific reports* **2**, 1002
